# A hurdle and negative binomial model approach to analyzing the gender differences in diagnostic imaging utilization under high-deductible health plans

**DOI:** 10.3389/fpubh.2025.1476782

**Published:** 2025-03-27

**Authors:** Qingyu Hu, Sarah Zheng

**Affiliations:** ^1^School of Economics and Business Administration, Chongqing University, Chongqing, China; ^2^Gustavson School of Business, University of Victoria, Victoria, BC, Canada

**Keywords:** high-deductible health plans, negative binomial regression, hurdle model, genders, diagnostic imaging services

## Abstract

**Introduction:**

High-Deductible Health Plans (HDHPs) have been increasingly adopted as a cost-containment strategy in healthcare. However, their impact on the utilization of diagnostic imaging services, particularly across different genders, remains underexplored. This study explores how HDHPs enrollment affect imaging utilization rates and usage patterns among patients of different genders, and it examines the associated gender disparities across various imaging modalities.

**Methods:**

Using data from the 2010 Thomson-Reuters MarketScan Commercial Database, we conducted a quantitative analysis employing Negative Binomial Regression and Hurdle models. The models assessed the association between HDHPs enrollment and diagnostic imaging utilization, with a focus on gender-based differences in usage patterns.

**Results:**

The analysis revealed that males generally utilize diagnostic imaging services less frequently than females. After HDHPs enrollment, overall imaging utilization declined by 7%, with a more pronounced reduction observed among male enrollees. Specifically, the likelihood of initial ultrasound utilization among males dropped by 8.2% more than among females. However, once at least one imaging procedure had been initiated, gender differences in utilization among HDHPs enrollees were no longer significant.

**Discussion:**

The findings suggest that HDHPs have a gender-differentiated effects on diagnostic imaging utilization, with males experiencing a more significant reduction, especially in the initial use of diagnostic imaging and in the use of ultrasound services. These results highlight the need for gender-sensitive approaches in health insurance policy design and emphasize the importance of targeted patient education to promote equitable access and resource allocation.

## 1 Introduction

In recent years, the medical imaging market has experienced significant growth, particularly among populations covered by Medicare Part B and private insurance (1–4). Recent data indicate that the U.S. medical imaging market reached approximately $10.03 billion in 2022 and is projected to grow at a compound annual growth rate (CAGR) of 5.5% in the coming years (5). Additionally, the diagnostic imaging services market was valued at $130.38 billion in 2023 and is expected to expand to $206.84 billion by 2030, reflecting a continued rise in imaging expenditures and utilization (6). As a crucial component of modern medicine, diagnostic imaging technologies—such as Magnetic Resonance Imaging (MRI), Computed Tomography (CT), Positron Emission Tomography (PET), Ultrasound (US), and X-ray—play a pivotal role in early disease screening and precision medicine, significantly contributing to clinical decision-making. Currently, imaging procedures account for approximately 10% of total healthcare expenditures (1, 7). However, the rapid expansion of diagnostic imaging has also raised concerns about overutilization, with some imaging procedures classified as low-value care, especially in the context of defensive medicine, where overdiagnosis and overtreatment have become growing areas of concern (8–14). Studies estimate that 20–50% of imaging procedures may be unnecessary (15), leading to increased healthcare costs and exposing patients to unnecessary treatments and follow-up procedures (9). With increasing attention on optimizing the allocation of imaging resources within the healthcare system, examining the utilization patterns of diagnostic imaging and the factors influencing its use—particularly the role of health insurance policies—has become a critical issue in contemporary healthcare policy and practice.

In recent years, the U.S. healthcare system has implemented various policy measures to control the low-value utilization of diagnostic imaging, such as Prior Authorization. However, the latest data indicate that imaging utilization rates remain high (5, 6). Studies suggest that 20–50% of imaging examinations may be unnecessary (15), partly due to healthcare professionals’ insufficient awareness of imaging referral guidelines (16). Against this backdrop, research focus has gradually shifted from physician incentives to patient cost-sharing mechanisms (2, 17). As one of the fastest-growing health insurance products in the U.S., High-Deductible Health Plans (HDHPs) are designed to reduce unnecessary healthcare utilization by increasing patients’ out-of-pocket costs (18, 19). Relevant literature has demonstrated that higher cost-sharing leads to a corresponding decline in healthcare utilization (20–22). Zheng et al. (17) found that HDHPs significantly reduce both diagnostic imaging utilization and healthcare expenditures. Other studies have reported that HDHPs make patients less willing to undergo breast imaging (23). However, in emergency departments, research by Chou et al. (24) indicates that while HDHPs significantly reduce overall emergency department utilization, they do not have a significant impact on the use of low-value imaging once a patient has decided to seek emergency care. Additionally, some studies have found that HDHPs do not significantly affect specific screening procedures, such as Transcranial Doppler screening (25). These findings suggest that while HDHPs effectively reduce overall diagnostic imaging utilization, their impact varies substantially across different patient populations.

In response, HDHPs have undergone multiple adjustments over the past two decades and have witnessed significant transformations in the U.S. healthcare system, including the implementation of the Affordable Care Act (ACA). Following the passage of the ACA, HDHPs eliminated out-of-pocket costs for X-rays to encourage women to undergo mammography screening. However, studies have found that X-rays are merely the first step in breast cancer screening, and when further imaging requires out-of-pocket expenses, the completion rate of the screening process significantly declines (23, 26). To further optimize the coverage of HDHPs, in 2019, the U.S. Department of the Treasury and the Internal Revenue Service (IRS) expanded the scope of preventive healthcare benefits included in HDHPs by adding 14 chronic disease treatment drugs and services eligible for pre-deductible coverage, allowing patients to access necessary medical care even before meeting their deductible threshold (27). In 2020, the Trump administration implemented Notice 2020–15, allowing HDHPs to waive out-of-pocket costs for COVID-19 testing and treatment, which significantly alleviated the financial burden on individuals for medical expenses during the pandemic (28).

Despite multiple optimizations of HDHPs, some perspectives argue that the coverage of the HDHPs Plus program remains insufficient, particularly in terms of gender-specific healthcare needs (17, 29–31). Existing research indicates that although men generally have higher socioeconomic status, they face a greater risk of chronic diseases and a higher premature mortality rate (32–34). For example, among heart disease patients under the age of 65, nearly 75% are male (35); moreover, among the ten most common infectious diseases in the United States, men have a higher incidence rate than women in seven of them (36). These gender disparities may stem from differences in healthcare utilization between men and women and may be further influenced by health insurance policies (37–40). Kozhimannil et al. (41) found that the reduction in emergency department visits due to HDHPs was more significant among men. However, the impact of HDHPs on gender differences in diagnostic imaging utilization remains unclear. Therefore, further investigation into gender disparities among HDHPs enrollees in the use of diagnostic imaging, uncovering the mechanisms through which HDHPs affect different genders, and ensuring that patients of all genders can access diagnostic imaging services in a high-value manner have become pressing concerns and a key policy priority.

Existing literature suggests that under HDHPs, men are more likely to reduce healthcare utilization to lower costs, potentially leading them to delay or forgo necessary diagnostic imaging examinations (42–46). Additionally, HDHPs may induce a “Blunting Effect” on male imaging utilization, meaning that the reduction in healthcare services is particularly pronounced among men during the early stages of policy implementation but stabilizes over time (41). When analyzing the impact of HDHPs on healthcare utilization, existing studies primarily employ traditional Ordinary Least Squares (OLS) or Logistic regression models, among others (20, 47). However, these methods struggle to effectively address common issues in healthcare utilization data of overdispersion and zero inflation (48, 49). To address these limitations, this study employs Negative Binomial Regression and the Hurdle model to more precisely capture the impact of HDHPs on diagnostic imaging utilization across genders (50). Negative Binomial Regression is well-suited for handling overdispersed count data, particularly when the variance of healthcare utilization exceeds its mean, allowing for a more accurate modeling of medical service usage patterns (51). The Hurdle model is particularly appropriate for analyzing the two-stage decision-making process of imaging utilization (47, 52). The first stage employs Logit regression to assess whether there is a significant gender difference in the initial decision to undergo imaging, while the second stage utilizes Zero-Truncated Negative Binomial Regression to evaluate whether there are significant gender differences in subsequent imaging utilization after the first examination. This methodological approach provides a more comprehensive understanding of healthcare decision-making by accounting for both gender disparities in the initial imaging decision and subsequent imaging utilization.

Building on the above analysis, this study focuses on the gender differences in diagnostic imaging utilization under HDHPs and explores the following key research questions: First, does HDHP enrollment have differential effects on the utilization rates of diagnostic imaging services among male and female patients? Second, under the HDHP system, do males and females exhibit significant differences in their initial decision to undergo diagnostic imaging? Third, does gender influence further utilization of diagnostic imaging services after the initial examination among HDHP enrollees? Fourth, are there gender differences in the impact of HDHPs on the utilization of different types of imaging modalities, such as MRI, CT, US, and X-ray? Employing negative binomial regression and the Hurdle model, this study conducts an in-depth analysis of gender heterogeneity in diagnostic imaging utilization among HDHP enrollees. By addressing these questions, this research aims to fill the existing gap in the literature and provide empirical evidence to support the optimization of future health insurance policies.

## 2 Materials

### 2.1 Data and analytic sample

We rely on the 2010 Thomson-Reuters MarketScan Commercial Claims and Encounters database, which contains enrollment information for employer-sponsored private health plans, clinical utilization records, and gender classification data for 45,239,752 enrolled individuals. The year 2010 represents a critical policy juncture, as the U.S. healthcare system was undergoing significant reforms under President Obama’s leadership, marking the early phase of Affordable Care Act (ACA) implementation. This provides a unique policy context for examining the role of HDHPs during this period and their differential impact on diagnostic imaging utilization across genders.

Considering that young adults aged 18–20 are typically covered under their parents’ health insurance plans, particularly following the implementation of the ACA, the trend of dependent coverage has become more prominent (18). This implies that their healthcare decisions and insurance choices are likely influenced by family financial status and parental decisions rather than autonomous selection. Therefore, from these individuals, we selected a cohort of 31,405,163 adults aged 21 to 64 years; we also focused on those with consistent health plan enrollment records throughout the year without any changes in the type of health plan, amounting to 22,026,367 individuals; furthermore, we excluded individuals without diagnostic codes and those with missing geographic location data, resulting in a final sample size of 21,440,466.

### 2.2 Variable definitions

We conducted a detailed analysis of each participant involved in the study. The primary focus was on the various diagnostic imaging examinations they underwent, which included computed tomography (CT), magnetic resonance imaging (MRI), positron emission tomography (PET), ultrasound (US), and X-ray inspections. These data were obtained by analyzing inpatient and outpatient claim records, employing Current Procedural Terminology (CPT) codes as the standard. To comprehensively understand each member’s use of imaging studies, we compiled the total number of examinations they received.

Our study observed that the distribution of imaging exams exhibited significant heavy-tailed characteristics. To adjust for outliers in the data, we applied the 99.99th percentile Windsorization method in our analysis (53, 54), which involved replacing all data values above the 99.99th percentile with the value at the 99.99th percentile, thereby ensuring the accuracy and representativeness of the analysis.

In this research, we defined the ‘Diagnostic Imaging Utilization’ as the primary dependent variable, representing the total number of diagnostic imaging services the participants received in 2010. This directly reflects the overall demand for and frequency of use of imaging services by the participants within a specific period. Additionally, we created a binary indicator to determine whether each participant had undergone at least one imaging exam in 2010. With this data, we gained insights into the utilization patterns of imaging services, which facilitated the exploration of the association between HDHPs and the volume of diagnostic imaging services used and the gender differences therein.

Our prominent independent variables included two binary variables: gender and whether the participant was enrolled in a High-Deductible Health Plans (HDHPs). The gender variable aids researchers in analyzing and understanding the differences in the utilization of diagnostic imaging services between males and females. The definition of an HDHPs follows the standards set in 2010, which stipulated a minimum annual deductible of 1,200 for individual coverage and 2,400 for family coverage (55). Non-HDHPs were defined as enrollment in Exclusive Provider Organizations (EPO), Health Maintenance Organizations (HMO), Point of Service (POS), Preferred Provider Organizations (PPO), or capitated POS plans. The selection of this independent variable is aimed at analyzing the impact of HDHPs enrollment on diagnostic imaging services among patients of different genders, particularly exploring behavioral changes and differences in healthcare service utilization among participants of different genders when faced with higher out-of-pocket expenses.

For the independent variables, in addition to the HDHPs Enrollment Status, we also introduced an interaction term between gender and HDHPs Enrollment Status. By analyzing this interaction term, we can reveal the potential differences in behavior and tendencies in the use of diagnostic imaging services between males and females under the context of HDHPs. Such analysis is instrumental in understanding how gender factors contribute to the utilization of healthcare services, particularly when dealing with insurance plans with high deductible amounts.

Control variables in this study included participants’ age, geographic location, and health status. Age is an important control variable as the demand for diagnostic imaging services may vary significantly across different age groups. Geographic location was also considered because the distribution of medical resources and usage habits in different regions may influence the utilization of imaging services. Lastly, we used binary indicators for 127 Hierarchical Condition Categories (HCCs) as proxies to capture the detailed diagnostic profile of individuals. These 127 HCCs were generated by processing medical claims data using the Health and Human Services-Hierarchical Condition Categories (HHS-HCC) software (56, 57) including conditions such as Diabetes without complication, Major depressive and bipolar disorders, Asthma, and so on. By controlling for these variables, the study could minimize the confounding effects of other factors and more accurately assess the gender differences in the impact of HDHPs on the utilization rate of diagnostic imaging.

## 3 Methods

To examine whether HDHP enrollment affects the utilization rate of diagnostic imaging services differently across genders, we employed the Negative Binomial Regression model for empirical analysis (50). Since healthcare utilization data is typically count data and often exhibits overdispersion (i.e., variance exceeding the mean), the Negative Binomial Regression model effectively addresses this dispersion issue, ensuring more robust estimations. This study applies the standard Negative Binomial Regression model, with its detailed mathematical formulation available in Chapter 3 of Cameron & Trivedi (50). The dependent variable is the total number of diagnostic imaging services each sample patient underwent in 2010, while the key independent variables include HDHP enrollment status (HDHPs Enrollment), gender (Gender), and their interaction term (Gender × HDHPs Enrollment).

To investigate whether there are gender differences in the initial decision and further imaging utilization of diagnostic imaging under the HDHPs system, we employed the Hurdle model for analysis. Given that medical utilization data often contain a large number of zero values (i.e., some patients did not undergo any imaging examinations), traditional count regression models may lead to estimation bias. The Hurdle model, with its two-stage structure, allows for a more precise modeling of medical decision-making processes. The specific mathematical formulation can be found in Chapter 4 of Cameron & Trivedi (50). The first stage of the Hurdle model utilizes a Logit regression to examine whether significant gender differences exist in the decision to undergo the first diagnostic imaging service under the HDHPs system. The dependent variable is a binary indicator of whether an individual received at least one diagnostic imaging service (1 = underwent imaging; 0 = did not undergo imaging). The primary independent variables include HDHPs enrollment status (HDHPs Enrollment), gender (Gender), and the interaction term between gender and HDHPs enrollment (Gender × HDHPs Enrollment). The second stage of the Hurdle model employs a Zero-Truncated Negative Binomial Regression to assess whether gender differences significantly affect further imaging utilization among those who have already undergone at least one imaging examination. The dependent variable is the total number of imaging procedures received by each patient. The independent variables remain the same as in the first stage, including HDHPs enrollment status, gender, and their interaction term. Since this stage only focuses on patients who have received at least one imaging examination, the dataset does not contain zero values. As a result, using a standard Negative Binomial Regression may introduce bias. Therefore, a Zero-Truncated Negative Binomial Regression model is employed to enhance the robustness of the estimates. By applying the Hurdle model, this study enables a deeper exploration of the mechanisms through which HDHPs impact different gender groups.

To investigate whether gender differences exist in the impact of HDHPs on different types of diagnostic imaging (MRI, CT, US, X-ray, etc.), we employed the first-stage Logit regression of the Hurdle model for empirical analysis. Our primary focus was on whether patients underwent a specific type of imaging examination rather than the total number of examinations. Therefore, a binary Logit regression was used to model the imaging selection patterns of different gender groups under the HDHPs system. Independent Logit regression models were separately constructed for five imaging modalities: MRI, CT, PET, US, and X-ray. The dependent variable was a binary indicator of whether a specific imaging examination was performed (0 = No, 1 = Yes). The primary independent variables included HDHPs enrollment status (HDHPs Enrollment), gender (Gender), and the interaction term (Gender × HDHPs Enrollment). This analysis allowed us to further assess whether HDHPs exert differential effects on male and female patients for various diagnostic imaging service.

All regression models controlled for patient age, the squared term of age (Age^2^), gender, geographic location (3-digit ZIP Code), and 127 binary Hierarchical Condition Categories (HCCs) to account for individual heterogeneity and reduce potential confounding bias. In the second stage of the Hurdle model, we further controlled for service setting (outpatient, inpatient, office, emergency room) and imaging modality (CT, MRI, PET, US, X-ray). These variables were not included in other models as they only applied to patients who had already undergone imaging examinations. Standard errors were reported across all regression analyses to ensure the robustness of the estimates. For the second-stage Zero-Truncated Negative Binomial Regression of the Hurdle model, we reported estimated regression coefficients. For the first-stage Logit regression of the Hurdle model, we reported average marginal effects, with standard errors calculated using the Delta method (58). All analyses were conducted using SAS 9.3 and Stata 12. A *p*-value threshold of <0.001 was set to ensure statistical significance.

## 4 Results

The descriptive statistics of the sample data are presented in Table 1. The total sample includes 21,440,466 individuals, of whom 47.43% are male and 52.67% are female. In Table 1, the *p* < 0.001 for the Female, indicating significant gender differences between HDHPs enrollees and non-enrollees, which is a focus of our research. Moreover, the proportion of HDHPs enrollees was significantly higher in the North Central region (33.52%) compared to other areas, and the average age of HDHPs enrollees was slightly lower than that of non-HDHPs enrollees (43.48 vs. 44.00). The HCC risk scores indicate that HDHPs participants were healthier than non-HDHPs participants. Among HDHPs enrollees, the top five disease tiers primarily involved diabetes without complication, major depressive and bipolar disorders, asthma, and so on, with a similar distribution of diseases among non-HDHPs enrollees. There were significant differences between the tiers (*p* < 0.001).

**Table 1:** HDHPs enrollees characteristics.

			Region (%)						Top 5 HHS-HCC hierarchies (%)				
	Number of HDHPs	Female	Northeast	North Central	South	West	Average age	Average HCC risk scorez	Diabetes without complication	Major depressive and bipolar disorders	Average HCC risk scorez	Average HCC risk scorez	“Breast (age 50+) and prostate cancer, benign/uncertain brain tumors, and other cancers and tumors”
HDHPs	1,670,362	52.24%	14.43%	33.52%	35.65%	16.4	43.48	1.13	4.09	2.44	2.28	0.96	0.99
Others	19,770,104	52.71%	15.27%	23.84%	40.14	20.75	44	1.26	5.13	2.83	2.64	1.33	1.08
Total	21,440,466	52.67%	15.21%	24.59%	39.79	20.42	43.96	1.25	5.05	2.8	2.61	1.3	1.07
P	—	< 0.001	< 0.001	< 0.001			< 0.001	< 0.001	< 0.001	< 0.001	< 0.001	< 0.001	< 0.001

To further investigate the utilization of diagnostic imaging, we conducted a summary statistical analysis of various diagnostic imaging service utilization rates, with detailed results presented in Table 2. Among all sampled patients, 38.54% underwent at least one imaging examination. For those with at least one imaging examination, the average per person was 3.22 imaging studies, covering CT, MRI, PET, Ultrasound, and X-ray. Among HDHPs participants, X-rays had the highest usage rate (25.03%), with an average of 2.22 X-ray uses among those who had used X-rays at least once. Although CT was not the most frequently used imaging examination, patients who initially utilized CT scans did so on average 2.52 times per person. The lowest usage rate among all imaging studies was for PET (0.28%). There were significant differences in the initial use of five types of diagnostic imaging among participants. Non-HDHPs participants showed the same trend but with higher usage rates.

**Table 2:** Summary statistics on imaging utilization.

% & Average no.	CT	MRI	Ultrasound	PET	xray	Any imaging
HDHPs	7.65% (2.52)	6.75% (1.44)	13.61% (2.10)	0.28% (1.50)	25.03% (2.22)	36.51% (3.11)
Others	8.76% (2.50)	7.50% (1.46)	14.19% (2.11)	0.30% (1.49)	27.04% (2.28)	38.71% (3.23)
Total	8.68% (2.50)	7.44% (1.46)	14.19% (2.11)	0.30% (1.49)	26.88% (2.28)	38.54% (3.22)
P	<0.001 (<0.001)	<0.001 (0.001)	<0.001 (0.162)	<0.001 (0.819)	<0.001 (<0.001)	<0.001 (<0.001)

The Full Model column of Table 3 presents the empirical results of the negative binomial regression model. From the results of the Negative Binomial Regression model, it is evident that after controlling for participants’ age, geographic location, and 127 HCCs, HDHPs enrollees exhibited a statistically significant 7% reduction in imaging utilization rates compared to non-HDHPs enrollees. Notably, there exists a significant gender disparity in overall diagnostic imaging utilization rates, with males being 46% less likely to utilize imaging services than females. However, within the HDHPs-enrolled population, males further reduced their imaging utilization rates by an additional 1% compared to females, despite their already lower baseline utilization. This finding suggests that HDHPs exert a stronger suppressive effect on diagnostic imaging utilization among male participants.

**Table 3:** Effect of HDHPs on imaging utilization between males and females.

Imaging utilization analysis			
Model	Full model	Hurdle Model	
	Negative binomial	Logit	Zero-truncated negative binomial
Male	−0.46^***^(0.00)	−0.55^***^(0.00)	−0.09^***^(0.00)
1 = HDHPs/CDHP = 1	−0.07^***^(0.00)	−0.08^***^(0.00)	−0.01^***^(0.00)
Male*1 = HDHPs/CDHP = 1	−0.01^***^(0.00)	−0.01^***^(0.00)	−0.00(0.00)
CT			0.70***(0.00)
MRI			0.48***(0.00)
PET			0.26***(0.00)
US			0.54***(0.00)
x-rays			0.56***(0.00)
Observations	21,440,466	21,440,454	8,263,204
Pseudo *R*^2^	0.045	0.095	0.174

The Hurdle Model column of Table 3 presents the empirical results of the hurdle model. In the first stage of the Hurdle model, logit regression results indicate that HDHPs enrollees had a significantly lower probability of utilizing any diagnostic imaging services, a finding that aligns with Zheng et al. (17). This stage primarily investigates whether there exists a significant gender disparity among patients who undergo diagnostic imaging for the first time. The results show that, across all study participants, males had a 1% significantly lower likelihood of undergoing their first diagnostic imaging examination compared to females. This finding suggests that HDHPs may reinforce preexisting gender differences in healthcare utilization behaviors. The second stage of the Hurdle model, utilizing Zero-Truncated Negative Binomial Regression, further examines whether there exists a significant gender disparity in subsequent imaging utilization among those who have already undergone at least one diagnostic imaging service. The results indicate that once HDHPs enrollees have received at least one diagnostic imaging service, the gender difference in further utilization is no longer significant. This suggests that HDHPs primarily impact the initial decision to undergo diagnostic imaging, whereas after the first examination, the frequency of subsequent imaging utilization does not differ significantly by gender.

To further explore whether there are significant gender differences in the impact of HDHPs on the utilization rates of different diagnostic imaging services, we conducted an empirical analysis using the first-stage Logit regression of the Hurdle model, with the results presented in Table 4. The findings indicate that male HDHPs enrollees were 1.2% less likely than female enrollees to undergo any imaging examination for the first time. This suggests that HDHPs may amplify gender disparities in medical behavior, healthcare needs, and cost sensitivity, leading to a higher likelihood that males, compared to females, avoid or delay their initial imaging examinations under the HDHPs system. Among all types of diagnostic imaging services, the impact of HDHPs was most significant for CT, MRI, and US utilization, with the largest gender difference observed in US usage. Specifically, male HDHPs enrollees were 8.2% less likely than female enrollees to undergo US examination for the first time. This finding suggests that, based on the pre-existing gender disparities in diagnostic imaging utilization, male HDHPs enrollees are more likely to reduce their use of US services. Notably, although male patients under HDHPs generally utilized imaging services less frequently than female patients, their initial utilization rates of MRI and X-ray were 2.8 and 1.9% higher, respectively, than those of female patients. Among these modalities, X-ray was the most frequently used imaging service, which may be attributed to its relatively lower cost and broad applicability.

**Table 4:** Effect of HDHPs on different type of imaging utilization.

	CT	MRI	US	PET	xray	All
Male	−0.209***	−0.199***	−1.380***	−0.0573***	−0.192***	−0.551***
	(−120.13)	(−109.84)	(−844.75)	(−5.92)	(−177.78)	(−550.29)
1 = HDHPs/CDHP = 1	−0.133***	−0.109***	−0.0108***	0.0103	−0.0954***	−0.0809***
	(−31.09)	(−24.65)	(−3.62)	−0.45	(−35.98)	(−33.55)
Male*1 = HDHPs/CDHP = 1	0.00681	0.0283***	−0.0818***	0.00201	0.0192***	−0.0124***
	(−1.06)	(−4.26)	(−13.66)	−0.06	(−4.94)	(−3.46)
Observations	21,440,427	21,440,450	21,440,450	21,433,499	21,440,446	21,440,446

**Figure 1:**
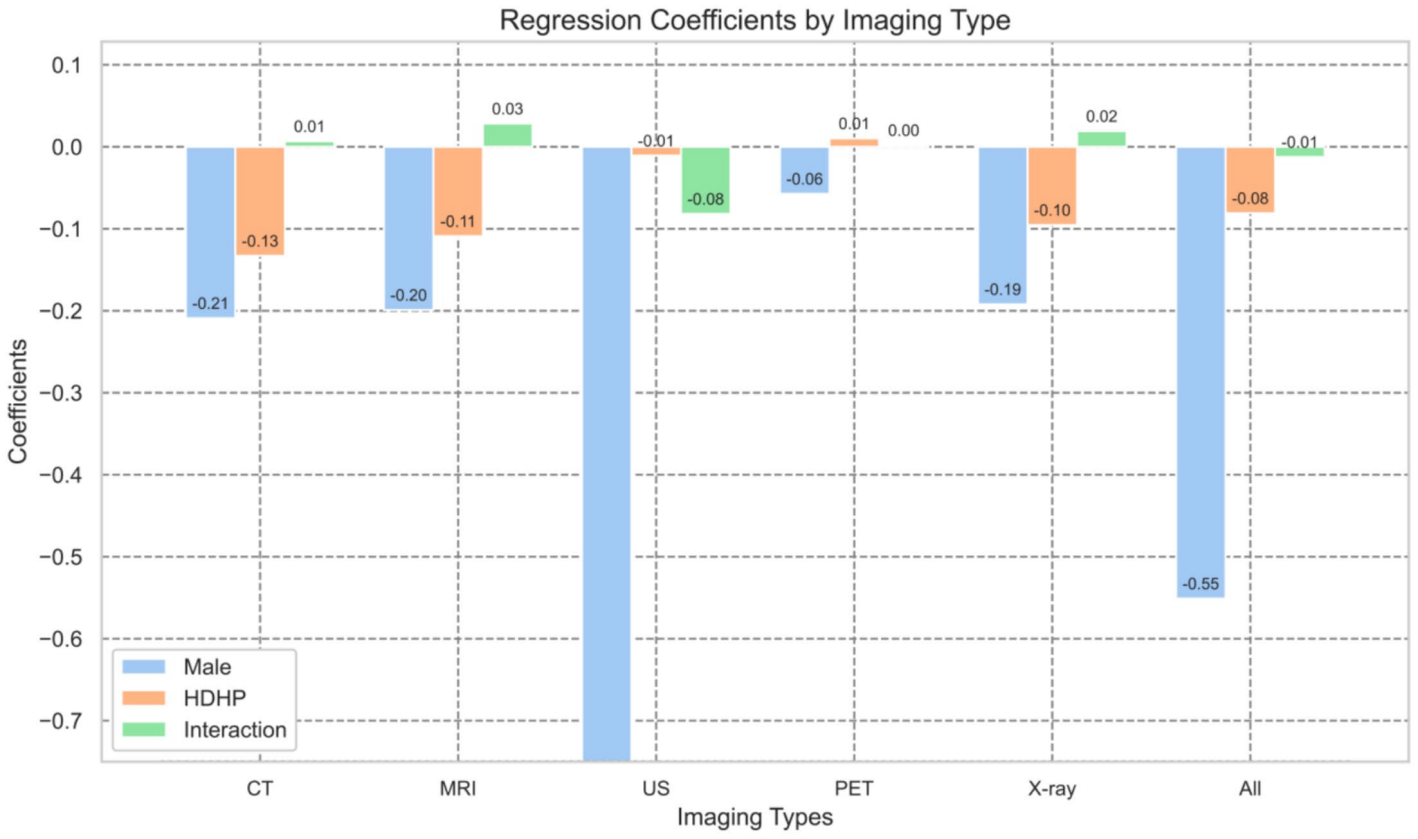
Regression coefficients by different imaging types.

**Figure 2:**
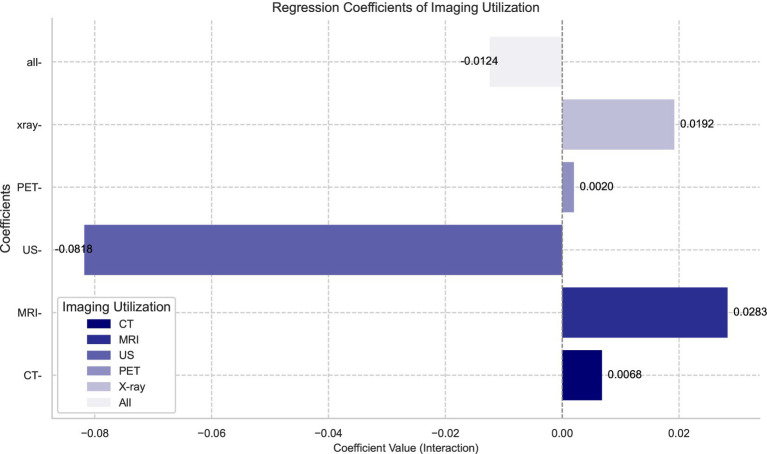
Gender differences in different imaging types.

To more intuitively illustrate the impact of HDHPs on different types of diagnostic imaging services and their gender disparities, we plotted the regression coefficients for various imaging modalities based on Table 4, as shown in Figures 1, 2. Figure 1 compares the regression coefficients of three key variables: gender (the decline in utilization rates among males compared to females), HDHPs enrollment status (HDHP), and their interaction term. From the blue bars in Figure 1, it is evident that significant gender differences exist in the utilization rates of diagnostic imaging services, with males generally exhibiting lower utilization rates. Following the implementation of HDHPs, we observed a notable overall decline in diagnostic imaging utilization, as indicated by the orange bars. This finding further indicates that the introduction of HDHPs has reduced patients’ utilization of diagnostic imaging services, particularly among males.

The reduction in diagnostic imaging utilization due to the introduction of HDHPs also exhibits distinct trends across genders, which is more clearly illustrated in Figure 2. Figure 2 primarily focuses on the gender disparities among HDHPs enrollees (interaction term). Notably, in the case of ultrasound (US) examinations, although males initially had lower utilization rates, the introduction of HDHPs significantly amplified this gender disparity. Among HDHPs enrollees, males’ utilization of US services was 8.2% lower than that of females. This phenomenon is primarily due to the relatively lower demand for ultrasound among males, while its application is more prevalent in gynecological and obstetric examinations. Furthermore, as out-of-pocket expenses increase, males tend to reduce the use of such services when considering both cost and necessity (42–46).

## 5 Conclusion and discussion

HDHPs are designed to encourage enrollees to reduce unnecessary medical utilization, such as imaging examinations with lower clinical diagnostic efficacy, while ensuring the appropriate use of preventive and essential healthcare services. Existing studies have shown that HDHPs reduce patients’ utilization of healthcare services (59–61), which aligns with our findings. However, previous research has rarely focused on the differential impact of HDHPs on patients of different genders, potentially overlooking the distinct behavioral patterns exhibited by different gender groups in response to the HDHP mechanism. Therefore, after controlling for age, geographic location, and health status, this study further explores the differential effects of HDHPs enrollment on the utilization rate of diagnostic imaging among male and female patients, providing a more comprehensive understanding of its policy implications. Our key findings are as follows: (1) There are significant gender differences in HDHPs enrollment (see Table 1), and the impact of HDHPs enrollment varies significantly across different types of diagnostic imaging (see Table 2). (2) Compared to non-HDHPs enrollees, HDHPs enrollees experienced an overall reduction of 7% in diagnostic imaging utilization, with a more pronounced decrease among male enrollees (see Table 3, Full Model). (3) Under the HDHPs system, there are significant gender differences in the initial decision to undergo diagnostic imaging. However, once the decision to undergo imaging is made, the frequency of subsequent imaging utilization no longer exhibits significant gender differences (see Table 3, Hurdle Model). (4) The impact of HDHPs on the first-time utilization rates of different types of diagnostic imaging exhibits significant gender disparities. Specifically, male HDHPs enrollees experienced a significantly greater reduction in the first-time utilization rate of ultrasound (US) compared to female enrollees, with a decline of 8.2% (see Table 4).

Based on the main findings of this study, we conducted the following discussion and analysis.

First, our study reveals gender differences in the utilization patterns of diagnostic imaging services. Based on the HDHPs enrollment status and imaging utilization rates among different gender groups (Tables 1, 3), we find significant gender disparities in both HDHPs enrollment and diagnostic imaging usage. After the introduction of HDHPs, men are more likely to reduce or delay imaging examinations, particularly when making the initial decision on whether to undergo diagnostic imaging (Logit regression in the Hurdle Model column of Table 3). This finding suggests that pre-existing gender differences in diagnostic imaging utilization may be exacerbated by HDHPs, underscoring the importance of incorporating gender considerations into health insurance design. However, once patients decide to undergo an imaging examination, the gender disparity disappears (Zero-Truncated Negative Binomial regression in the Hurdle Model column of Table 3), indicating that HDHPs primarily influence the initial decision-making stage rather than subsequent imaging utilization. This further highlights the objectivity of medical decision-making: the influence of high deductibles on gender differences is most pronounced at the initial decision-making stage. However, once a diagnosis is made and a treatment plan is established, patients tend to rely more on their physicians for subsequent decisions based on their specific medical conditions and clinical needs. As a result, the impact of high deductibles on continued healthcare utilization diminishes.

Secondly, our study reveals the impact of HDHP enrollment on the utilization of different types of diagnostic imaging and the associated gender disparities. Table 2 presents the utilization rates and average number of imaging examinations across different insurance statuses. The results indicate significant differences in diagnostic imaging utilization between HDHP enrollees and non-enrollees. Among all imaging modalities, X-ray was the most frequently used, with a utilization rate of 25.03% among HDHP enrollees and 27.04% among non-HDHP enrollees, a statistically significant difference (*p* < 0.001). This suggests that HDHP enrollment significantly reduces the use of X-ray imaging. Furthermore, Table 4 shows that male HDHP enrollees experienced a significantly greater reduction in the use of ultrasound (US) compared to females, with an 8.2% decline in first-time US utilization. In contrast, the first-time utilization rates for MRI and X-ray among male HDHP enrollees increased by 3 and 2%, respectively. This trend may be attributed to gender-specific healthcare needs. For example, females are more likely to require ultrasound examinations, such as obstetric ultrasound for pregnancy monitoring and gynecological ultrasound for conditions like ovarian cyst evaluation. As a result, the impact of HDHPs on ultrasound utilization among female patients is less pronounced. Conversely, male HDHP enrollees are more likely to reduce non-urgent imaging examinations, such as US, and instead opt for MRI and X-ray, which have higher clinical necessity for male health conditions. Specifically, MRI plays a crucial role in male health screenings, such as prostate MRI, and in the assessment of musculoskeletal injuries, including joint and spinal injuries. Meanwhile, X-ray remains an essential diagnostic tool for men due to its lower cost and widespread application in detecting common health issues such as fractures and pulmonary diseases.

Finally, our study sheds light on the potential impact of HDHPs on men’s healthcare utilization patterns. As shown in Table 3, we found significant gender differences in healthcare utilization behaviors under HDHPs, with male patients exhibiting a more pronounced response to HDHP enrollment. While previous research has not specifically examined the impact of transitioning to HDHPs on male and female diagnostic imaging utilization rates, extensive qualitative and exploratory studies have consistently documented that men generally have lower healthcare utilization rates than women (37–40). This phenomenon is largely attributed to the cultural construct of masculinity, which emphasizes independence and discourages seeking medical help. Population-based studies further suggest that men often view avoiding healthcare services as a way to reinforce their sense of autonomy and self-reliance (2, 14, 35, 36, 62). As indicated by the first-stage results of the Hurdle Model in Table 3, in the first year following the transition to HDHPs, the utilization of any diagnostic imaging services significantly declined among male enrollees, with a greater reduction than that observed in females. This finding suggests that high-level cost-sharing mechanisms may exert additional influence on male patients, potentially exacerbating their tendency to delay or forgo necessary medical examinations. Such delays may heighten the risk of diagnoses and worsen health outcomes. Additionally, as shown in Table 4, we observed a general decline in US utilization among HDHP enrollees, with a more pronounced reduction among male participants. This trend reflects pre-existing gender differences in healthcare decision-making, which HDHP enrollment may further amplify. Given these findings, clinicians can play a critical role in mitigating this effect. They should enhance awareness of how changes in health plans influence healthcare utilization, particularly among male patients. Furthermore, healthcare providers should leverage clinical interactions to emphasize the importance of timely medical care, thereby reducing healthcare delays driven by financial considerations.

Building on the above discussion and analysis, this study proposes a series of policy recommendations to assist policymakers in developing more gender-sensitive and inclusive HDHP guidelines. First, HDHPs should implement a more inclusive insurance design by refining cost-sharing mechanisms to better accommodate gender-based differences in healthcare utilization, particularly in the use of diagnostic imaging services. This approach aims to mitigate accessibility barriers and disparities in medical decision-making resulting from gender-based differences. Second, HDHPs should establish more reasonable deductible structures tailored to specific medical needs. For example, women, who are more likely to require obstetric ultrasounds and mammograms, should be provided with lower deductibles or full coverage for these essential screenings. Conversely, men may be more likely to forgo necessary imaging procedures such as prostate MRIs due to cost concerns. To address this, HDHPs should introduce lower or zero-deductible options for such examinations, encouraging timely screenings and reducing potential health risks. Third, we recommend enhancing health education initiatives for HDHP enrollees, particularly focusing on medical decision-making interventions for men. Strengthening awareness and guidance can help mitigate care avoidance driven by financial considerations and promote more informed and timely healthcare utilization.

Regarding future research directions, we propose several key areas for further exploration. First, further research is needed to examine how HDHPs shape the utilization patterns of various healthcare services beyond diagnostic imaging, particularly across different gender groups. Second, future studies should evaluate the long-term health consequences of HDHPs, with a focus on whether delayed initial diagnostic decisions lead to heightened health risks and increased medical expenditures. Additionally, further research could explore the structural variations in HDHPs, such as the inclusion of health savings accounts (HSA) and health reimbursement arrangements (HRA) (63, 64), and their differential effects on healthcare utilization among male and female patients. These insights would help refine the design of HDHPs to enhance their inclusivity and effectiveness.

## Data Availability

The data analyzed in this study is not publicly available and is subject to the following licenses/restrictions: The Center for Applied Studies in Health Economics (CASHE) holds a renewable license for The MarketScan Commercial Claims Database that allows the data to be used by Penn State researchers and students. Requests to access these datasets should be directed to cashe@psu.edu.
